# RdDM-Associated Chromatin Remodelers in Soybean: Evolution and Stress-Induced Expression of CLASSY Genes

**DOI:** 10.3390/plants14162543

**Published:** 2025-08-15

**Authors:** Paula Machado de Araújo, Arthur Gruber, Liliane Santana Oliveira, Sara Sangi, Geovanna Vitória Olimpio, Felipe Cruz Paula, Clícia Grativol

**Affiliations:** 1 Laboratório de Química e Função de Proteínas e Peptídeos, Centro de Biociências e Biotecnologia, Universidade Estadual do Norte Fluminense Darcy Ribeiro, Campos dos Goytacazes 28013-602, RJ, Brazil; paulaaraujo.bio@gmail.com (P.M.d.A.); geovannavop@gmail.com (G.V.O.); felipe.cpaula64@gmail.com (F.C.P.); 2 Departamento de Parasitologia, Instituto de Ciências Biomédicas, Universidade de São Paulo, São Paulo 05508-000, SP, Brazil; argruber@usp.br (A.G.); liliane.sntn@gmail.com (L.S.O.); 3 Laboratório de Biologia Celular e Tecidual, Centro de Biociências e Biotecnologia, Universidade Estadual do Norte Fluminense Darcy Ribeiro, Campos dos Goytacazes 28013-602, RJ, Brazil; sarasm.sangi@gmail.com

**Keywords:** epigenetic regulation, RNA-directed DNA methylation, CLSY1-4, phylogeny, profile hidden Markov models, *Glycine max*

## Abstract

RNA-directed DNA methylation (RdDM) is an epigenetic mechanism involved in several biological processes in plants, requiring complex machinery including the chromatin remodeling protein CLASSY (CLSY). The CLSY family regulates global and locus-specific DNA methylation and was initially identified in *Arabidopsis thaliana*. Despite reports in other plants, detailed knowledge about CLSY proteins in soybean is scarce. In this work, we used profile hidden Markov models (profile HMMs) specifically constructed for CLSY detection to identify new members in soybean and to analyze their phylogenetic relationships across bryophyte, basal angiosperm, basal eudicot, monocots, and eudicots. We identified two new candidates for CLSY1-2 and one for DRD1 in soybean and, for the first time, detected CLSY and DRD1 genes in *Aquilegia coerulea*. Phylogenetic analysis indicated two main CLSY groups: one similar to *Arabidopsis* CLSY1-2 and another to CLSY3-4. Gene duplication analysis demonstrated that whole-genome duplication/segmental duplication events contributed to CLSY family expansion in soybean. RT-qPCR analysis showed that CLSY and five other epigenetic regulator genes had stress-modulated expression during soybean germination under salt and osmotic stress, with variation among cultivars. Our findings enhance comprehension of the evolutionary dynamics of the CLSY family and furnish insights into their response to abiotic stress in soybean.

## 1. Introduction

Epigenetic marks are characterized by changes in genomes that do not alter the primary DNA sequence, and that can be inherited through cell division [1]. Epigenetic pathways in plants, including DNA methylation, histone modification, and small RNA-guided DNA methylation, contribute to phenotypic plasticity and survival under unpredictable environmental conditions, and can act individually or together to promote tolerance to biotic and abiotic stresses [2,3]. Also, epigenetic regulation plays an important role in various stages of plant development, such as seed development, germination, fruit ripening, and sexual and asexual reproduction [4,5,6]. In fact, epigenetic modifications provide an additional level of genetic regulation which impacts the growth, environmental adaptation, and the evolutionary history of plants [2,7].

RNA-directed DNA methylation (RdDM) is an epigenetic pathway, unique to plants, in which small RNAs (sRNAs) guide de novo DNA methylation [8]. RdDM was first reported in transgenic tobacco plants infected with viroids, consisting of a circular non-coding RNA [9]. RdDM is involved in the response to biotic stresses, such as those caused by bacteria, viruses, and fungi [8,10], and abiotic stresses, such as salt [11] and heat stress [12].

Another important function of RdDM is the repression of transposable element (TE) activity. Without TE silencing by the RdDM pathway, TEs can be inserted into genes or promoters, which can affect gene expression or cause mutations in proteins [8]. Therefore, RdDM helps maintain genomic stability [13], especially in plants with high TE content, such as maize, where approximately 85% of the genome is composed of TE [14]. Furthermore, the RdDM pathway contributes to the regulation of stress-induced TEs activation. One example is the retrotransposon *ONSEN*, which is upregulated during heat stress in *Arabidopsis*, but is repressed by sRNAs associated with RdDM [15].

RdDM can direct DNA methylation to cytosines in all sequence contexts, i.e., CG, CHG, and CHH, where H represents any nucleotide excluding G [10]. In plants, RdDM is the only pathway that adds de novo DNA methylation to unmethylated regions. The canonical RdDM pathway involves two main processes: the biogenesis of small interfering RNAs (siRNAs), and DNA methylation at target loci in DNA [8,10]. Initially, the RNA Polymerase IV (Pol IV) interacts with the chromatin remodeler CLASSY 1 (CLSY1) and the SAWADEE HOMEODOMAIN HOMOLOG 1 (SHH1), and forms a complex that binds to heterochromatin. Pol IV transcribes short single-stranded RNAs (ssRNAs), about 30 to 45 nucleotides (nt) in length, which are converted to double-stranded RNAs (dsRNAs) by RNA-DEPENDENT RNA POLYMERASE 2 (RDR2) associated with Pol IV. dsRNAs are cleaved into 24-nt siRNAs by the endoribonuclease DICER-LIKE 3 (DCL3). Then, the 24-nt siRNAs are methylated at the 3′ end by HUA ENHANCER 1 (HEN1) and incorporated into ARGONAUTE 4 or 6 (AGO4, AGO6) proteins. The AGO-sRNA duplex binds to complementary RNA transcribed by RNA Polymerase V (Pol V), and recruits the DNA methyltransferase DOMAINS REARRANGED METHYLTRANSFERASE 2 (DRM2), which methylates nearby DNA [8,10,16].

The CLSY1 protein is characterized by the presence of the SNF2 and Helicase C domains, and acts together with RDR2 and NRPD1a, a subunit of Pol IVa, in the production of 24-nt siRNAs [17]. Besides CLSY1, the CLSY family comprises three other members called CLSY2, CLSY3, and CLSY4 [18]. The four CLSYs are required for global and locus-specific regulation of DNA methylation. In locus-specific regulation, different chromatin modifications occur to produce 24-nt siRNAs, depending on which CLSY proteins are involved. CLSY1 and CLSY2 are required for the association between SHH1 and the Pol IV complex, in a manner dependent on histone H3 lysine 9 (H3K9) methylation. In the production of 24-nt siRNAs at loci controlled by CLSY3 and CLSY4, CG methylation is required [19]. A study has shown that, in addition to acting in the canonical RdDM pathway, the four CLSY proteins mediate DNA demethylation at specific loci, demonstrating the dual role that the CLSY family plays in balancing methylation and demethylation reactions [20]. Furthermore, other research revealed that CLSY1-4 can control tissue-specific DNA methylation patterns in *Arabidopsis*. Tissues with different CLSY expression levels had distinct DNA methylation patterns. For example, the four CLSY genes were expressed in flower buds, CLSY3 exhibited strong expression in ovules, and CLSY1 was expressed in leaves and rosettes. These findings reveal that locus-specific regulation in conjunction with tissue-specific expression of CLSYs generates epigenetic diversity during plant development [21].

Although CLSY genes have been investigated in *Arabidopsis*, few studies have reported their presence, function, and diversification in other plant species, particularly in legumes such as soybean (*Glycine max*). Soybean is an important crop worldwide, belonging to the Fabaceae family. It is considered an ancient polyploid, as a result of duplications that occurred approximately 59 and 13 million years ago. For this reason, almost 75% of the genes of this species occur in multiple copies [22]. Soybean is also regarded as a model legume plant for genomic studies, both in basic and applied research [23]. However, the characterization of CLSY genes in soybean remains largely unknown. After CLSY genes were described in *Arabidopsis* [17], they were also reported in rice [24], maize [25], chickpea [26], grapevine [27], and grasses [28]. Recently, the phylogenetic relationship of CLSY homologs between land plants and charophytic green algae was also analyzed [29]. The only mention of CLSY genes in soybean to date is found in the study by DiBiase et al. (2024) [30], who investigated the epigenetic responses, particularly DNA methylation, of two soybean lines, one resistant and one susceptible, to the pathogen *Phytophthora sansomeana*. The authors cited five CLSY genes upregulated following inoculation, among 79 genes involved in the RdDM pathway [30]. However, the research is not focused on this gene family, but rather on the expression analysis of several genes in a context of biotic stress in soybean. Therefore, detailed information on the CLSY family in soybean remains limited, particularly regarding its evolution, phylogenetic relationships, and response to abiotic stresses. Here, we built CLSY-specific profile HMMs to investigate CLSY members in soybean and different plant species, and show their phylogenetic relationships. We also analyzed the expression of a potential CLSY gene during soybean germination under abiotic stress conditions. These data will contribute to the knowledge of the structural characteristics, evolutionary relationships, and expression patterns of CLSYs in soybean.

## 2. Results and Discussions

### 2.1. Construction of Specific Profile HMMs of CLSY Proteins

**Figure 1:**
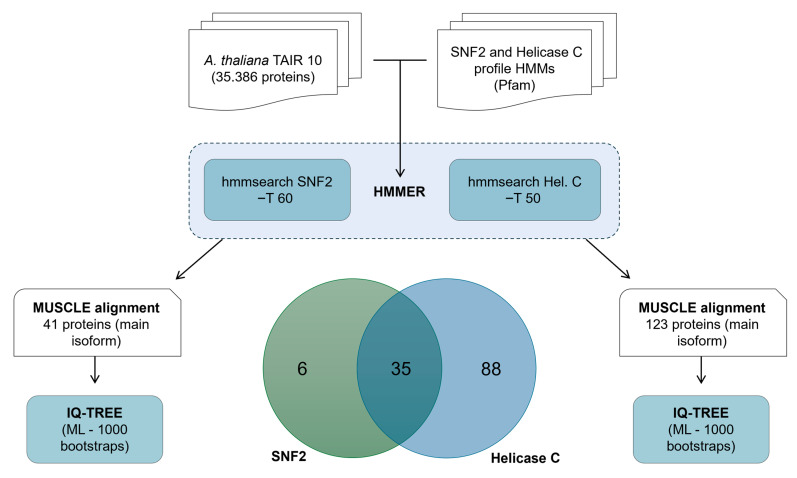
Workflow of the protein search approach using profile HMMs of SNF2 and Helicase C in *Arabidopsis*, followed by phylogenetic analysis. Protein sequences were retrieved using hmmsearch with Pfam profile HMMs (parameters: −T 60 for SNF2 and 50 for Hel_C), aligned with MUSCLE, and used to construct phylogenetic trees in IQ-TREE (maximum likelihood, 1000 bootstrap pseudoreplicates).

The identification of CLSY members in plant genomes is challenging, as this family of proteins contains two widespread domains—SNF2 and Helicase C [17]. Most of the studies describing CLSY genes have used pairwise alignment tools, such as BLAST, to identify novel sequences from sequencing data [26,28,29,30]. However, this method may not be sensitive enough to detect remote homologs. In this sense, profile-based alignment methods, such as profile HMMs, improve the ability to detect and classify sequences [31,32]. To identify CLSY members in plants, we constructed specific profile HMMs using CLSYs from *Arabidopsis*. Initially, all 35,386 *Arabidopsis* protein sequences were downloaded from Phytozome v13. To identify CLSY proteins in this dataset, we used hmmsearch (HMMER package) with Pfam-derived profile HMMs of SNF2 and Helicase C profile HMMs. In total, 41 proteins containing the SNF2 domain and 123 proteins with the helicase C domain were found. Of these, 35 proteins contained both domains (Figure 1).

The proteins from each domain and those having both domains were aligned using MUSCLE and then utilized to generate phylogenetic trees in IQ-TREE (Figures S1–S3). In the resulting trees, the four CLSY proteins of *Arabidopsis*, CLSY1 (AT3G42670), CLSY2 (AT5G20420), CLSY3 (AT1G05490), and CLSY4 (AT3G24340), are highlighted in red. The tree of 41 proteins containing the SNF2 domain (six proteins with solely the SNF2 domain and 35 containing both) presented a monophyletic group with the four CLSYs (Figure S1). The tree with the 123 proteins featuring the Helicase C domain (88 proteins containing only the Helicase C domain and 35 with both) also exhibited a monophyletic group of CLSY proteins (Figure S2). Figure S3 also displays a phylogenetic tree of 35 proteins that contain both SNF2 and Helicase C domains. Two additional trees were also generated, the former using sequences covering only the 41 SNF2 and excluding the Helicase C domain of the 35 proteins with both domains (Figure S4), and the latter using 123 Helicase C domains, excluding the SNF2 domain of the 35 proteins (Figure S5). The tree derived from the SNF2 domain, without the Helicase C domain (Figure S4), showed no difference in the topology compared to the tree using both domains (Figure S1), with the four CLSYs remaining in a monophyletic group. This result suggests that these CLSY proteins are monophyletic when analyzed only from the point of view of the SNF2 domain. However, in the tree constructed exclusively with Helicase C domain sequences, the four CLSY proteins were divided into three different groups, with only the CLSY1 and CLSY2 proteins in a single clade (Figure S5). This indicates that CLSY proteins are paraphyletic when analyzed solely using the Helicase C domain. Together, these results suggest that the CLSY protein group became differentiated from other proteins containing the SNF2 domain and that the Helicase C domain was subsequently incorporated in distinct events.

CLSY-specific profile HMMs were constructed with individual executions of TABAJARA using protein datasets of the 41 complete proteins shown in the tree of Figure S1, 123 complete proteins represented in Figure S2, and 41 proteins containing solely the SNF2 domain (Figure S4), respectively. The profile HMMs generated from each dataset were validated for the detection of the four *Arabidopsis* CLSY proteins. Models derived from each dataset were concatenated and used with the HMM-Prospector program to interrogate different plant protein datasets for the detection of novel CLSY proteins.

### 2.2. Identification, Phylogenetic Relationship, and Structural Analysis of CLSY Family in Plants

The specific profile HMMs detected corresponding proteins in 11 of the 12 plant species analyzed. We ran the HMM-Prospector with –rc (reduce cutoffs) parameter set up to 1.0, 0.8, and 0.6 for all 12 plant genomes. Next, we compared the resulting proteins in each dataset to verify the best cutoff. Using 0.6 reduced cutoff, we found a total of 56 putative CLSY proteins from the following organisms: moss *Physcomitrella patens* (Pp-1); basal angiosperm *Amborella trichopoda* (AmTr-2); basal eudicot *Aquilegia coerulea* (Aqcoe-6); monocots *Brachypodium distachyon* (Bradi-6), *Oryza sativa* (LOC_Os-6), *Sorghum bicolor* (Sobic-5), and *Zea mays* (Zm-5); and dicots *Arabidopsis thaliana* (AT-6), *G. max* (Glyma-9), *Phaseolus vulgaris* (Phvul-6), and Vitis vinifera (VIT-4). The search for CLSY proteins in the unicellular alga *Chlamydomonas reinhardtii* did not generate significant results based on the criteria used. Similarly, a phylogenetic analysis showed that other components of the RdDM pathway are absent in *C. reinhardtii*, such as NRPD1, DRM2, and DEFECTIVE IN RNA DIRECTED DNA METHYLATION 1 (DRD1), a member of the DDR complex involved in the Pol V pathway [33]. A study that investigated methylation patterns in plants and animals found that *C. reinhardtii* has unusual methylation patterns, and probably has different mechanisms to flowering plants [34].

**Figure 2:**
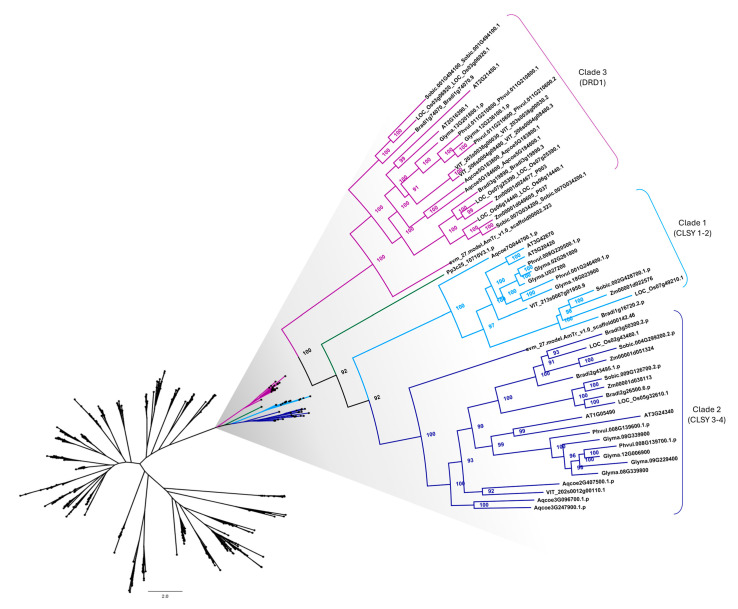
Phylogenetic tree of proteins containing the SNF2 and Helicase C domains from 11 species, highlighting the monophyletic group containing the CLSY and DRD1 proteins. IQ-TREE software (version 2.2.5) was used to construct the tree utilizing the maximum likelihood method. The values showed on the branches represent the bootstrap values from 1000 pseudoreplicates. Light blue, dark blue, and purple represent clades 1, 2, and 3, respectively. The single *P. patens* protein is shown in green. The complete tree can be found in the Supplementary Materials (Figure S6).

To better understand the phylogenetic relationship of CLSY family members, a phylogenetic tree was generated with 447 proteins containing the SNF2 and Helicase C domains from the 11 species, including the 56 putative CLSY proteins (Figure S6). We observed a monophyletic group containing three clades: clade 1, with 13 proteins, including CLSY1 and CLSY2 from *Arabidopsis*, represented in light blue; clade 2, with 22 proteins, containing *Arabidopsis* CLSY3 and CLSY4, displayed in dark blue; clade 3, with 20 proteins, including the other two proteins found in *Arabidopsis* (AT2G16390 and AT2G21450) that are not classified as CLSY, represented in purple; and an outlier protein from *P. patens* (Pp3c25_10710V3.1), shown in green (Figure 2).

Clade 1, which includes CLSY1-2, presents nine of the 11 species analyzed, with no proteins from the species *P. patens* and *A. trichopoda*. In clade 2, which contains CLSY3-4, 10 of the 11 plant species are present, with only *P. patens* missing. The occurrence of CLSY family members from basal angiosperm, basal eudicot, monocots, and eudicots indicates the presence of this family throughout plant evolution.

Clade 3 comprises proteins from the DRD1 family, a subfamily of SNF2 chromatin-remodeling proteins, which also harbor the Helicase C domain [35]. Yang and collaborators (2018) [20] showed that the protein AT2G16390, classified as DRD1, forms a clade with AT2G21450 and with the three *O. sativa* proteins (LOC_Os03g06920, LOC_Os06g14440, and LOC_Os07g25390) contained in clade 3. CLSY1-4 from *Arabidopsis* and DRD1 formed a monophyletic group with proteins from the SNF2 gene family, which indicates that CLSY and DRD1 proteins are closely related [20]. Besides, *Arabidopsis* CLSY1 is considered a homologue of DRD1 [17]. Taking all these results into account, it is feasible to assume that a monophyletic group containing CLSY and DRD1 proteins could be formed, as shown in Figure 2.

A single *P. patens* protein was an outlier of the other three clades. It has already been reported that some components of the RdDM pathway are absent in *P. patens*, such as RNA-DIRECTED DNA METHYLATION 1 (RDM1), which is part of the DDR complex as well as DRD1 [33]. The absence of specialized Pol V pathway proteins, which facilitate the de novo methylation step, may indicate that *P. patens* has a separate pathway of Pol V evolution or represents an interrupted early stage before acquiring components existing in flowering plants [33].

When comparing the results obtained using the profile HMM-based alignment method with pairwise BLAST alignments reported in the literature, we found that the profile HMM approach identified a greater number of CLSY proteins in some species. In soybean, the genes Glyma.02G261800 and Glyma.U027200, CLSY1-2 candidates, and Glyma.12G236100, DRD1 candidate, were identified using profile HMMs, but were not reported in the data obtained by BLAST [30]. The same occurred with the common bean (*P. vulgaris*) genes Phvul.001G246400.1 (CLSY1-2 candidate), Phvul.008G139600.1 and Phvul.008G139700.1 (CLSY3-4 candidates), and Phvul.011G210800.1 (DRD1 candidate), which were not mentioned in the study that analyzed CLSYs in land plants, including *P. vulgaris* [29]. Similarly, in *B. distachyon*, the gene Bradi2g43495.1 (CLSY3-4 candidate) was not included among the genes of this species identified by BLAST [28]. Additionally, through profile HMMs, we also identified six putative CLSY/DRD1 members in *A. coerulea*: one CLSY1-2, three CLSY3-4, and two DRD1. Until now, there was no description of these gene families in the literature for this species. These data suggest that the use of profile HMMs offers greater sensitivity in detecting members of the CLSY and DRD1 gene families compared to traditional pairwise alignment methods such as BLAST.

The gene ID, gene length, number of exons, protein length, presence of other domains, and chromosomal location of the identified proteins were compiled and are listed in Table 1. The length of the genes varied from 1938 to 15,347 base pairs (bp). The number of exons ranged from 1 to 12. The protein length varied from 405 to 1875 amino acids (aa). Only three proteins presented other domains. The Glyma.18G023900 and Pp3c25_10710V3.1 proteins exhibited the SAWADEE domain, and the LOC_Os07g49210.1 protein showed the Methyltransferase domain.

**Figure 3:**
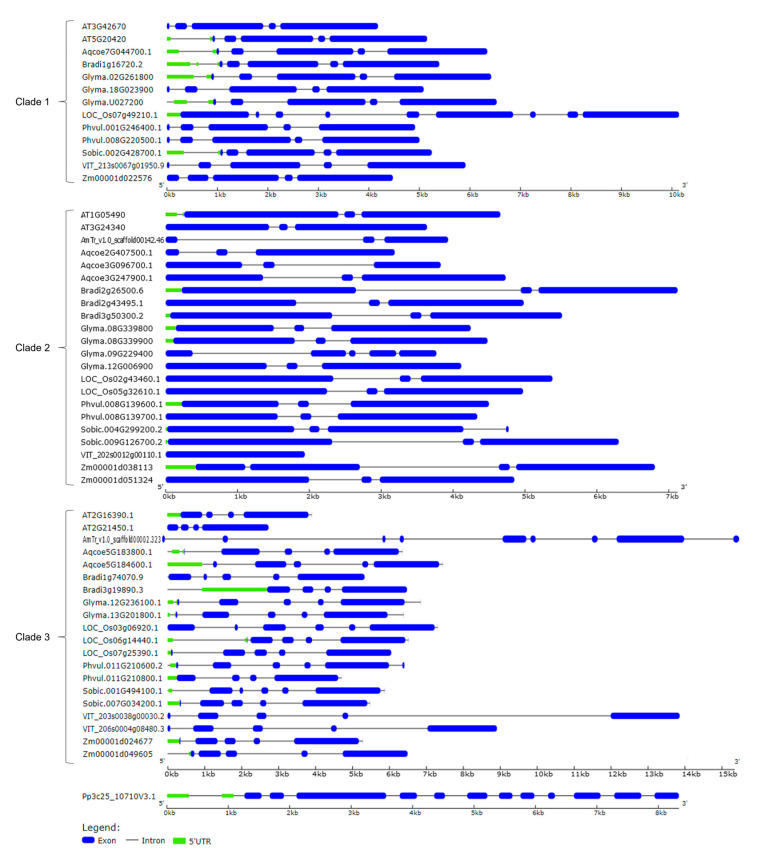
Gene structure of CLSYs, considering the clade division shown in the phylogenetic tree. Exons and introns are represented by blue boxes and black lines, respectively. The gene structure was generated based on the coding sequence and UTR regions of each gene using the GSDS 2.0 tool. The length of exons and introns is indicated in kilobases (kb).

We also analyzed intron-exon patterns to understand the structural diversity of CLSY genes. As shown in Figure 2, CLSY family members can be divided into two clades (clades 1 and 2) considering the division of *Arabidopsis* CLSY1-4. In the first clade, 12 of 13 genes had five exons, with gene lengths ranging from 4288 to 7454 bp. Only the LOC_Os07g49210.1 gene from *O. sativa* had nine exons and a size above 10 kb. In the second clade, 18 of 22 genes had three exons, and in the remaining four genes the number of exons varied from 1 to 5. The genes in clade 3, possibly related to the DRD1 family, showed a greater variation in the number of exons compared to clades 1 and 2, ranging from 4 to 9 exons. The single *P. patens* gene showed the highest number of exons among all clades, with 12 exons (Figure 3). In general, members of the same clade presented similar exon-intron structures. This observation reinforces the division of clades shown in the phylogenetic analysis.

**Figure 4:**
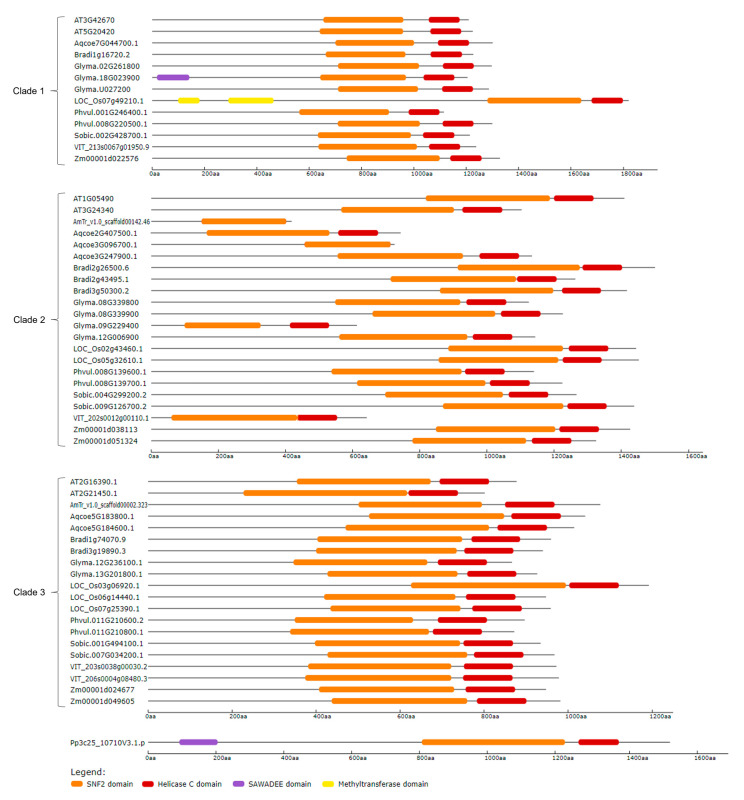
Protein domain architecture following the clade division from the phylogenetic analysis. Domains were predicted using the Pfam database. The size of proteins can be estimated from the scale in number of amino acids (aa). The corresponding color of each domain is indicated in the legend: SNF2 in orange, Helicase C in red, SAWADEE in purple, and Methyltransferase in yellow.

To understand the domain architecture of CLSY members, their protein structures were analyzed (Figure 4). Almost all proteins presented both SNF2 and Helicase C domains, except for the proteins AmTr_v1.0_scaffold00142.46 and Aqcoe3G096700.1 from clade 2, which did not present the Helicase C domain. Three proteins showed other domains besides SNF2 and Helicase C. The proteins Glyma.18G023900 from clade 1 and Pp3c25_10710V3.1 presented the SAWADEE domain, while LOC_Os07g49210.1 from clade 1 presented the Methyltransferase domain. These two additional domains are possibly involved in RdDM pathway processes. In a general context, the similarity between the gene and protein structures of members of clade 1 with CLSY1-2 of *Arabidopsis*, as well as members of clade 2 with CLSY3-4 indicates that proteins from these two clades potentially integrate the CLSY family.

**Figure 5:**
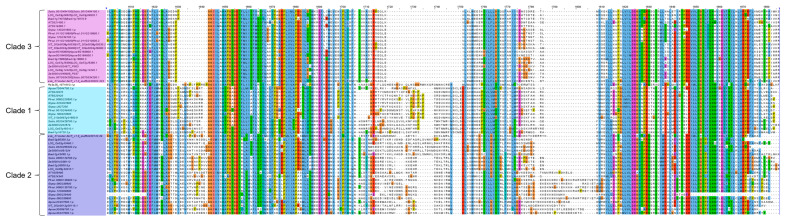
Multiple sequence alignment of the SNF2 domain obtained in Jalview, highlighting clades 1, 2, and 3. The clades are in the order in which they were arranged in the phylogenetic tree (Figure S6). A color scale for amino acids was used to indicate sequence similarity. Hydrophobic residues are in blue, positively charged in red, negatively charged in magenta, polar in green, cysteines in pink, glycines in orange, prolines in yellow, and aromatic residues in cyan. Gaps indicate unconserved regions.

The multiple alignment of the SNF2 domains of the 56 CLSY proteins showed a group of amino acid residues conserved only among CLSY3–4, but absent in CLSY1–2 and the other subfamily (Figure 5). This observation supports the result presented in the phylogenetic tree in Figure S4, which shows that *Arabidopsis* CLSYs form a unique clade when only the SNF2 domain is used as a parameter. In contrast, the Helicase C domain is conserved among the different species in the three clades (Figure S7), which also reinforces what was shown in the phylogenetic tree containing only the Helicase C domains from *Arabidopsis* (Figure S5), where the CLSYs were not a monophyletic clade. These data demonstrate the importance of the SNF2 domain for the identification of CLSY proteins.

### 2.3. Duplication Events of CLSY Genes in Soybean

Soybean was the species with the most genes identified in the three clades, with three genes in clade 1, four genes in clade 2, and two genes in clade 3. To further understand the expansion of soybean genes related to the CLSY family, we analyzed the duplication events of the genes included in clades 1 and 2, as well as the genes phylogenetically close to DRD1 in clade 3. Almost all genes were duplicated through whole-genome duplication (WGD)/segmental events, except Glyma.08G339900, which underwent a tandem duplication (Table 2). This suggests that WGD/segmental duplication played an important role in the expansion of soybean genes related to the CLSY and DRD1 families. WGD events correspond to complete duplication of chromosomes, and segmental duplications indicate long stretches of duplicated sequences with high identity. Tandem duplications produce a copy of an adjacent gene, generating tandemly arrayed genes [36]. Studies have shown that, in the soybean genome, the predominant type of duplication is segmental [37,38], which is consistent with our results.

Considering that soybean underwent two rounds of WGD [22], we analyzed the collinear relationships of the duplicate pairs and identified ten pairs of paralogs that have close phylogenetic relationships (Table 2). All soybean genes in clade 1 are paralogs to each other, and the same is true for genes in clades 2 and 3. The nonsynonymous to synonymous substitutions ratio values (Ka/Ks) of the genes ranged from 0.239974 to 0.754383 and were used to estimate the selection pressure. The Ka/Ks ratio = 1 indicates neutral selection, while a Ka/Ks ratio of <1 corresponds to negative selection and a Ka/Ks ratio of >1 implies positive selection [39]. All Ka/Ks ratio values obtained were <1, which indicates that soybean paralogous genes are under purification or stabilization selection.

It was observed that the duplication date of the ten pairs of paralogs ranged from 9.03 to 47.6 Mya (Table 2). The paralog pair Glyma.02G261800/Glyma.U027200 showed the lowest Ka/Ks value (~0.24) and the shortest divergence time (9.03 Mya), which may indicate that this gene pair maintained its functions after duplication. The soybean genome underwent two WGD events, the first 59 Mya and the second 13 Mya [22]. Of the 10 paralog pairs, eight were duplicated after the first WGD event and two were duplicated close to the second event. Therefore, the two duplication events may have contributed to the expansion of these genes in soybean.

### 2.4. Tissue Expression Profile of CLSY Genes in Arabidopsis and Soybean

To analyze the expression profiles of the *Arabidopsis* genes identified in clades 1, 2, and 3, the tissues showing the highest expression of each gene in the ePlant database were selected. The tissues include the shoot apex, the seed at 24 h of imbibition, seed tissues at different stages of development, parts of the flower, and young seeds. The genes CLSY1 and AT2G16390 (DRD1) showed higher expression in the shoot apex transition and inflorescence tissues compared to the other genes. Only CLSY1 presented high expression in the 24 h imbibed seed. In developing seed tissues, in general, all genes showed higher expression in seed stages 4 to 7, except for CLSY3. The genes CLSY1, CLSY2, AT2G21450, and AT2G16390/DRD1 were highly expressed in the carpel of young flowers. CLSY3, CLSY4, AT2G21450 and AT2G16390/DRD1 exhibited greater expression in ovules and at different stages of young seeds (Figure S8). These observations demonstrate that there are similar expression patterns between the CLSYs and DRD1-related genes in different *Arabidopsis* tissues.

Data from different types of abiotic stresses show that the six *Arabidopsis* genes analyzed here can have altered expression profiles under adverse environmental conditions. Figure S8 lists nine types of stresses reported in shoot and root. In CLSY2, for example, after 30 min of osmotic stress by 300 mM mannitol, this gene is up-regulated in the shoot. After 3 h of the same treatment, the expression of this gene is lower in the shoot compared to the control (Figure S9). This demonstrates that CLSYs expression profiles can be modulated by abiotic stresses in *Arabidopsis*.

To investigate the expression profiles of soybean genes present in the three clades, RNA-seq data from the Soybean Expression Atlas database were used, relating to 14 tissues, including embryo, seed coat, seed, cotyledon, leaves, callus, nodule, pod, flower, hypocotyl, suspensor, shoot, endosperm and seedling (Figure S10). The gene Glyma.U027200 showed higher expression among tissues in comparison to the other genes, especially in the embryo tissue. We confirmed the expression profile of this gene in embryonic axes by RT-qPCR, compared to the gene Glyma.08G339900 (Figure S11). The paralogous gene pair Glyma.09G229400/Glyma.12G006900 presented a similar expression profile, showing expression only in the suspensor tissue. This may be indicative that these genes kept the same functions after the duplication. The other pairs of paralogs showed different expression levels in one or more tissues. This suggests that these genes may have acquired distinct functions after duplication.

**Figure 6:**
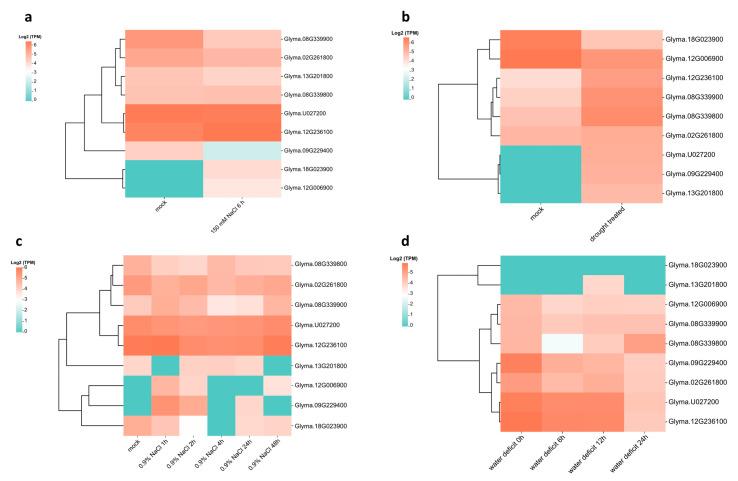
Log2 expression (TPM) of nine soybean genes from clades 1, 2, and 3 under stress conditions. Green and orange colors indicate low and high expression, respectively. (**a**) Soybean seedlings treated with 150 mM NaCl after six hours. (**b**) Soybean roots subjected to drought. (**c**) Soybean roots treated with 0.9% NaCl after 1, 2, 4, 24, and 48 h. (**d**) Soybean shoots subjected to water deficit after 0, 6, 12, and 24 h.

We also evaluated the expression profiles of soybean genes under salt and drought stress conditions by utilizing publicly available RNA-seq data (Figure 6). The expression of some of the analyzed genes was induced under different stress conditions. Treatment with 150 mM NaCl after six hours in seedlings induced the expression of the genes Glyma.18G023900 and Glyma.12G006900 (Figure 6a). In roots subjected to drought, almost all genes were induced, excluding only Glyma.18G023900 and Glyma.12G006900 (Figure 6b). Treatment with 0.9% NaCl in roots induced the expression of some genes, such as Glyma.09G229400 after 1, 2, and 24 h; and Glyma.12G006900 and Glyma.08G339900 after 1, 2, and 48 h (Figure 6c). In shoots subjected to water deficit, the Glyma.13G201800 gene was induced after 12 h, and the Glyma.08G339800 gene was induced after 24 h (Figure 6d). These data suggest that genes with induced expression may play an important role in the response to salt, drought, and water deficit stresses in soybean.

### 2.5. The Expression Profile of CLSY and Five Other Genes Involved in Epigenetic Regulation Can Be Modulated Under Abiotic Stresses During Soybean Germination

**Figure 7:**
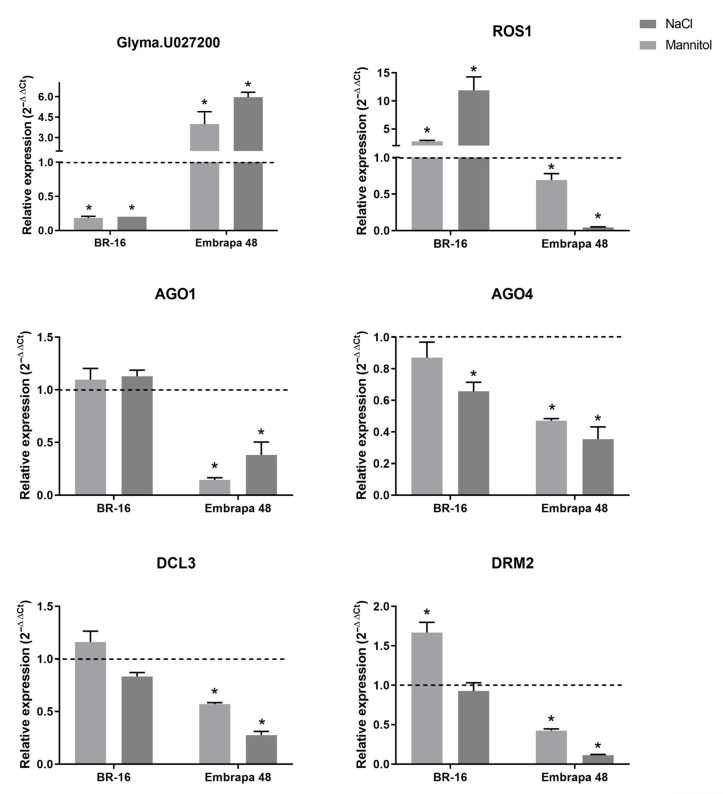
Expression profiles of the AGO1, AGO4, DCL3, DRM2, Glyma.U027200, and ROS1 genes during the germination of soybean seeds treated with 300 mM mannitol and 100 mM NaCl. The dashed line represents the expression of genes in control seeds. Asterisks indicate significantly different expression values between the control and treatments, according to the *t* test (*p* < 0.05).

Considering that the Glyma.U027200 gene showed higher expression among different soybean tissues in relation to the other genes, mainly in the embryo tissue (Figure S10), we investigated whether the expression of this gene could be altered under stress conditions in the embryonic axis during germination. For this, seeds of BR-16 (drought-sensitive) and Embrapa 48 (drought-tolerant) cultivars were treated with 100 mM NaCl and 300 mM mannitol to evaluate the effect of salt and osmotic stress on Glyma.U027200 gene expression. The embryonic axes of the seeds of both cultivars were removed after 30 h of treatment for RNA extraction and subsequent evaluation of gene expression by RT-qPCR. The Glyma.U027200 gene showed contrasting expression profiles between the two cultivars, being down-regulated in BR-16 in both treatments, and up-regulated in Embrapa 48, also under both stresses (Figure 7). A study that compared gene expression patterns in leaves and roots of BR-16 and Embrapa 48 under water deficit also showed different genes with contrasting expression between the cultivars. It was shown that Embrapa 48 presented 770 more up-regulated genes than BR-16. The RNA-seq data generated in this study showed that, for example, the gene Glyma.09G229400 (which appears in clade 2 of Figure 2) has a higher expression in the root of Embrapa 48 than in BR-16, under water deficit treatments of duration 100 and 150 min [40]. This indicates a possible genetic/molecular difference in the responses of these cultivars to stress.

In addition to the analysis performed for the gene Glyma.U027200 related to CLSY1-2, we also investigated the expression of five other genes involved in epigenetic regulation using the same treatments (Figure 7). The genes analyzed were the following: DRM2, AGO4, and DCL3, which are involved in the RdDM pathway; REPRESSOR OF SILENCE 1 (ROS1), responsible for removing DNA methylation; and AGO1, which participates in sRNA production in the non-canonical RdDM pathway [8]. In cultivar BR-16, the DCL3 and AGO1 genes showed no significant variation in expression compared to the control under both stresses. DRM2, on the other hand, exhibited higher expression under osmotic stress, and did not show variation under salt stress. ROS1 showed the highest expression in BR-16 under both stresses, especially in salt stress, while AGO4 was down-regulated in both treatments. In contrast, in Embrapa 48 all five genes were down-regulated in both mannitol and NaCl treatments. Therefore, among the genes analyzed, only the potential CLSY (Glyma.U027200) was up-regulated in Embrapa 48. As demonstrated in *Arabidopsis*, CLSY genes do not appear to follow the same expression patterns as other genes involved in DNA methylation. CLSY1-4 have the most diverse expression patterns in comparison to other components of the RdDM pathway, genes needed for the maintenance of DNA methylation, and demethylases [21]. This suggested that CLSYs may act with a different timing compared to other members of the RdDM pathway and could have an important role in Embrapa 48 epigenetic response during abiotic stress or in the epigenetic background in each cultivar.

The distinct expression patterns observed between the two cultivars reveal the molecular peculiarities inherent to the genotypes. Prior research has shown that Embrapa 48 exhibits a faster response to water stress than BR-16, modulating gene expression from the early stages of stress. This improved performance is supported by the up-regulation of transcription factor-related genes associated with drought, such as AP2 [40]. Due to the absence of traits associated with drought adaptability, the sensitive cultivar may exhibit a differential molecular response to drought stress in some cases [41]. Genes showing contrasting expression patterns between cultivars may be promising candidates for future studies aimed at enhancing plant stress tolerance [40].

Considering the role of stress-responsive transcription factors as regulators of gene expression, we analyzed the promoter region of the Glyma.U027200 gene, and identified binding sites for transcription factors involved in abiotic stresses in soybean. These included AP2 and WRKY, both associated with drought tolerance [42,43]; MYB and HD-Zip, which act under salt stress and dehydration/drought conditions [44,45]; NAC, which enhances salt stress tolerance [46]; and MADS-Box, related to the response to different abiotic stresses in plants, such as drought, salt, cold, heat, and oxidative stress [47]. These data indicate that this putative CLSY may be part of a regulatory network in response to adverse environmental conditions, especially under saline and drought stress.

## 3. Materials and Methods

### 3.1. Identification of CLSY Gene Family Members

Initially, we obtained the genomes of soybean and 11 additional plant species. The genomic and protein sequences of *A. trichopoda* v1.0, *A. coerulea* v3.1, *A. thaliana* TAIR10, *B. distachyon* v3.2, *C. reinhardtii* v5.5, *G. max* Wm82.a2.v1, *O. sativa* v7.0, *P. vulgaris* v2.1, *P. patens* v3.3, *S. bicolor* v3.1.1, *V. vinifera* v2.1, and *Z. mays* RefGen_V4 were obtained from Phytozome v13 (https://phytozome-next.jgi.doe.gov/) [48] (accessed on 6 May 2022). We used the profile hidden Markov models (profile HMMs) corresponding to the SNF2 (PF00176) and Helicase C (PF00271) domains available on the Pfam database (http://pfam.xfam.org/) [49] (accessed on 9 May 2022), to search for protein sequences with these profiles in the *A. thaliana* proteome. Next, we executed hmmsearch (HMMER package) with the parameters −T 60 and 50 for SNF2 and Hel_C profiles, respectively. The multiple sequence alignments of the resulting proteins were used as input for the program TABAJARA (Tool for Alignment Block Analysis Joining Appropriate Rational Approaches), available at https://github.com/gruberlab/TABAJARA (accessed on 11 August 2025), to construct specific profile HMMs for CLSY proteins. This program calculates position-specific information scores in a multiple sequence alignment and builds profile HMMs, identifying conserved and specific regions in biological sequences [32,50]. TABAJARA utilizes hmmsearch, hmmbuild and nhmmer from the HMMER package [51] and MUSCLE [52]. Three groups of sequences containing the SNF2 and Hel_C domains served as input for TABAJARA and the generated models were used with the HMM-Prospector program [32,50], available at https://github.com/gruberlab/hmmprospector (accessed on 11 August 2025), to detect CLSY proteins in the plant sequence datasets. The CLSY-specific profile HMMs constructed and used in this work are available as Supplementary Material (File S1).

### 3.2. Phylogenetic Analysis

Multiple alignments of protein sequences were performed using the MUSCLE tool [52]. The phylogenetic tree was constructed with the IQ-TREE software (version 2.2.5) [53] using the maximum likelihood method with 1000 pseudoreplicates. The model VT+F+I+G4 was automatically evaluated by ModelFinder [54] as the best-fit model. The phylogenetic tree was edited and visualized on Figtree v1.4.4 (http://tree.bio.ed.ac.uk/software/figtree/) (accessed on 13 May 2022).

### 3.3. Gene Structure and Domain Prediction

The gene structure was generated based on the coding sequence, 5′UTR, and 3′UTR of each gene in the Gene Structure Display Server (GSDS) 2.0 (http://gsds.gao-lab.org/) [55] (accessed on 8 August 2022). The prediction of the domains was performed in the Pfam database (http://pfam.xfam.org/) [49] (accessed on 9 May 2022). The domain sequences were aligned using MUSCLE with default parameters in the Jalview software [56], version 2.11.3.2.

### 3.4. Non-Synonymous and Synonymous (Ka/Ks) Analysis for Duplicated Pairs of CLASSY Genes in Soybean

Soybean genome sequences (*Glycine max* Wm82.a2.v1) along with GFF3 annotation files were obtained from the Phytozome v13 database [48]. The coding sequences (CDS) of CLSY genes were aligned based on the amino acid sequence orientations using ClustalW with its default settings [57]. Duplication events and collinearity relationships were identified using the Multiple Collinearity Scan toolkit (MCScanX) [58]. The non-synonymous (Ka) and synonymous substitution (Ks) rate were computed using KaKs_Calculator 3.0 [59] using the NG method [60]. The duplication date (million years ago, Mya) was estimated using the formula T = Ks/2λ × 10^–6^ Mya (λ = 6.5 × 10^−9^) [61].

### 3.5. Expression Analysis of CLSY Genes in Arabidopis and Soybean Tissues

The ePlant suite (https://bar.utoronto.ca/eplant/) [62] (accessed on 15 March 2023) was used to compare the expression patterns of CLSY genes already identified in *Arabidopsis*. To analyze the expression profiles of predicted CLSY genes in soybean tissues, the Soybean Expression Atlas v2 database was used (https://soyatlas.venanciogroup.uenf.br/) [63] (accessed on 23 March 2023). The expression of these genes was calculated in Transcripts Per Million (TPM), converted to log2, and visualized using heatmaps generated by the heatmap.2 function available in the gplots package in R [64], version 4.3.2.

### 3.6. Expression Analysis of a CLSY Gene During Soybean Germination Under Stress Conditions

Initially, the soybean seeds from cultivars BR-16 (drought-sensitive) and Embrapa 48 (drought-tolerant) were disinfected with 2% of sodium hypochlorite and then washed five times with sterilized distilled water [65]. Seeds were placed in Petri dishes (90 × 15 mm) with 12 mL of sterilized distilled water on germitest paper and treated with sodium chloride (NaCl) and mannitol at concentrations of 100 mM and 300 mM, respectively. For each treatment, two biological replicates were used, each comprising a pool of 10 seeds. Treated and untreated seeds were germinated in a biochemical oxygen demand (B.O.D.) incubator at 28 °C, without photoperiod [65,66]. At 30 h after imbibition (HAI), the radicles were manually sectioned for total RNA extraction using Trizol^®^ (Invitrogen, Waltham, MA, USA) according to the manufacturer’s instructions. The integrity of the RNA was verified by electrophoresis on 1% agarose gel stained with ethidium bromide. RNA quantification was carried out using a NanoDrop™ One spectrophotometer (Thermo Fisher Scientific, Waltham, MA, USA) [67]. The synthesis of cDNA was performed with 5 µg of RNA using the SuperScript™ III kit (Invitrogen, Waltham, MA, USA). The gene expression was evaluated by RT-qPCR with SYBR Green PCR Master Mix (Applied Biosystems, Waltham, MA, USA), using the StepOne™ Real-Time PCR System (Applied Biosystems, Waltham, MA, USA). Amplifications were performed in 48-well plates at a final volume of 10 μL. Each plate contained 1 μL of forward and reverse primers, 5 μL of SYBR Green, 2 μL of cDNA, and 2 μL of ultrapure water. Three technical replicates were used for each sample. The forward and reverse sequences of the primers used are listed in Table S1. The S-adenosylmethionine synthase 4 (METK4) gene was used as a constitutive reference gene for internal control [65]. Relative expression was calculated using the 2^−∆∆CT^ method [68]. The data obtained were submitted to the *t*-Test with a significance level of 95% (*p* < 0.05) in the GraphPad Prism v9.0 program (https://www.graphpad.com/) (accessed on 13 October 2023).

### 3.7. Promoter Sequence Analysis

To identify transcription factor binding sites in the promoter region of the Glyma.U027200 gene, we used the PlantPAN 4.0 tool (https://plantpan.itps.ncku.edu.tw/plantpan4/promoter_analysis.php, accessed on 11 August 2025) [69] (accessed on 31 July 2025). The 1000 bp upstream sequence from the start codon was retrieved from the genomic sequence available in the Phytozome v13 database [48].

## 4. Conclusions

Data about the phylogeny and evolution of the CLSY gene family in soybean are still incipient in the scientific literature. Furthermore, there is scarce information about CLSY expression profiles under environmental stress conditions. In our study, the construction of specific profile HMMs enabled a more sensitive and comprehensive analysis of CLSY proteins across soybean and other plant genomes. The proteins were divided into two clades, one containing 13 proteins, including CLSY1-2 from *Arabidopsis*, and the other containing 22 proteins, considering CLSY3-4 from *Arabidopsis*. From the analysis of the nonsynonymous to synonymous substitution ratio values (Ka/Ks), it was found that members of the CLSY family in soybean may have expanded from two WGD/segmental duplication events. Considering the high expression of the potential CLSY gene Glyma.U027200 in soybean embryos, we evaluated the expression of this gene in germination under salt and osmotic stress in the BR-16 and Embrapa 48 cultivars. The expression of the gene Glyma.U027200 and five other genes involved in different epigenetic regulatory mechanisms (DRM2, AGO4, DCL3, ROS1, and AGO1) showed contrasting expression profiles between the two cultivars. In addition to identifying novel putative CLSY and DRD1 genes not detected by pairwise alignment methods, this study provides the first report of these gene families in *A. coerulea*. Taken together, the results presented here provide valuable insights into the phylogeny, evolution, and gene expression profiles of CLSY family members, not only in soybean but also in other relevant plant species.

## Figures and Tables

**Table 1:** Gene identification and sequence characteristics of the 56 corresponding proteins identified from the profile HMMs.

Gene ID	Gene Length (bp)	Exons Number	Protein Length (aa)	Other Domains	Chromosome	Phylogenetic Tree Clade
Aqcoe7G044700.1	6524	5	1312	0	7	Clade 1
AT3G42670	4288	5	1257	0	3	
AT5G20420	5153	5	1262	0	5	
Bradi1g16720.2	5594	5	1261	0	1	
Glyma.02G261800	7254	5	1311	0	2	
Glyma.18G023900	5085	5	1236	SAWADEE	18	
Glyma.U027200	7454	5	1308	0	scaffold_265	
LOC_Os07g49210.1	10,327	9	1875	Methyltransferase	7	
Phvul.001G246400.1	4915	5	1179	0	1	
Phvul.008G220500.1	5002	5	1311	0	8	
Sobic.002G428700.1	5539	5	1231	0	2	
VIT_213s0067g01950.9	5914	5	1264	0	13	
Zm00001d022576	4684	5	1335	0	7	
AmTr_v1.0_scaffold00142.46	3931	3	405	0	scaffold00142	Clade 2
Aqcoe2G407500.1	3357	3	761	0	2	
Aqcoe3G096700.1	3828	3	719	0	3	
Aqcoe3G247900.1	5106	3	1173	0	3	
AT1G05490	4851	3	1411	0	1	
AT3G24340	3638	3	1133	0	3	
Bradi2g26500.6	8975	3	1507	0	2	
Bradi2g43495.1	6754	3	1286	0	2	
Bradi3g50300.2	6593	3	1416	0	3	
Glyma.08G339800	4517	3	1149	0	8	
Glyma.08G339900	7386	3	1247	0	8	
Glyma.09G229400	3769	5	618	0	9	
Glyma.12G006900	4114	3	1167	0	12	
LOC_Os02g43460.1	5383	3	1440	0	2	
LOC_Os05g32610.1	5705	3	1446	0	5	
Phvul.008G139600.1	4598	3	1143	0	8	
Phvul.008G139700.1	4337	3	1219	0	8	
Sobic.004G299200.2	5147	4	1279	0	4	
Sobic.009G126700.2	7022	3	1459	0	9	
VIT_202s0012g00110.1	1938	1	646	0	2	
Zm00001d038113	7648	4	1436	0	6	
Zm00001d051324	4853	3	1338	0	4	
AmTr_v1.0_scaffold00002.323	15,347	9	1095	0	scaffold00002	Clade 3
Aqcoe5G183800.1	8214	6	1060	0	5	
Aqcoe5G184600.1	8072	6	1027	0	5	
AT2G16390.1	3982	5	889	0	2	
AT2G21450.1	2947	4	817	0	2	
Bradi1g74070.9	5856	5	974	0	1	
Bradi3g19890.3	7475	4	948	0	3	
Glyma.12G236100.1	7244	6	884	0	12	
Glyma.13G201800.1	6578	6	954	0	13	
LOC_Os03g06920.1	7317	7	1198	0	3	
LOC_Os06g14440.1	7192	6	952	0	6	
LOC_Os07g25390.1	6671	5	967	0	7	
Phvul.011G210600.2	6631	6	901	0	11	
Phvul.011G210800.1	4894	5	872	0	11	
Sobic.001G494100.1	6270	6	946	0	1	
Sobic.007G034200.1	6488	6	971	0	7	
VIT_203s0038g00030.2	13,882	5	973	0	3	
VIT_206s0004g08480.3	8920	5	976	0	6	
Zm00001d024677	5604	6	951	0	10	
Zm00001d049605	7240	5	978	0	4	
Pp3c25_10710V3.1	8807	12	1534	SAWADEE	25	

**Table 2:** Paralog pair genes, Ka/Ks ratio values, and duplication type of soybean genes.

Gene ID	Gene ID	Ka	Ks	Ka/Ks	Duplication Date (Mya)	Selection Pressure	Duplication Type
Glyma.02G261800	Glyma.18G023900	0.223722	0.618747	0.361573	47.60	Purification or Stabilization selection	WGD or Segmental
Glyma.02G261800	Glyma.U027200	0.0281611	0.117351	0.239974	9.03	Purification or Stabilization selection	WGD or Segmental
Glyma.U027200	Glyma.18G023900	0.223407	0.614387	0.363626	47.26	Purification or Stabilization selection	WGD or Segmental
Glyma.08G339900	Glyma.08G339800	0.219666	0.373098	0.58876	28.70	Purification or Stabilization selection	Tandem
Glyma.08G339900	Glyma.09G229400	0.166574	0.407391	0.40888	31.34	Purification or Stabilization selection	WGD or Segmental
Glyma.08G339900	Glyma.12G006900	0.225172	0.396627	0.567	30.51	Purification or Stabilization selection	WGD or Segmental
Glyma.12G006900	Glyma.09G229400	0.131003	0.173655	0.754383	13.36	Purification or Stabilization selection	WGD or Segmental
Glyma.12G006900	Glyma.08G339800	0.14434	0.301214	0.47919	23.17	Purification or Stabilization selection	WGD or Segmental
Glyma.09G229400	Glyma.08G339800	0.14488	0.309337	0.468356	23.80	Purification or Stabilization selection	WGD or Segmental
Glyma.12G236100	Glyma.13G201800	0.09762	0.31009	0.3148	23.85	Purification or Stabilization selection	WGD or Segmental

## Data Availability

The original contributions presented in this study are included in the article/Supplementary Materials. Further inquiries can be directed to the corresponding author.

## Supplementary Materials

The following supporting information can be downloaded at: https://www.mdpi.com/article/10.3390/plants14162543/s1, Table S1: Primer sequences (forward/reverse) used in RT-qPCR analysis; Figure S1: Phylogenetic tree of 41 proteins containing the SNF2 domain. *Arabidopsis* CLSY1-4 proteins are highlighted in red. The values shown on the branches represent the bootstrap values from 1000 replicates; Figure S2: Phylogenetic tree with the 123 proteins featuring the Helicase C domain. *Arabidopsis* CLSY1-4 proteins are highlighted in red. The values shown on the branches represent the bootstrap values from 1000 replicates; Figure S3: Phylogenetic tree with the 35 proteins in common between the SNF2 and Helicase C domains. *Arabidopsis* CLSY1-4 proteins are highlighted in red. The values shown on the branches represent the bootstrap values from 1000 replicates; Figure S4: Phylogenetic tree containing the 41 SNF2 domains, but excluding the Helicase C domain of the 35 proteins with both domains. *Arabidopsis* CLSY1-4 proteins are highlighted in red. The values shown on the branches represent the bootstrap values from 1000 replicates; Figure S5: Phylogenetic tree comprising the 123 Helicase C domains, but excluding the SNF2 domain of the 35 proteins. *Arabidopsis* CLSY1-4 proteins are highlighted in red. The values shown on the branches represent the bootstrap values from 1000 replicates; Figure S6: Complete phylogenetic tree of the 447 identified proteins, with emphasis on the monophyletic group containing proteins related to the CLSY and DRD1 families. Clades 1, 2, and 3 are shown in light blue, dark blue, and purple, respectively. The values indicated on the branches represent the bootstrap values from 1000 replicates; Figure S7: Multiple sequence alignment of the Helicase C domain obtained in Jalview, highlighting the clades 1, 2, and 3. The clades are in the order in which they were arranged in the phylogenetic tree (Figure S6). A color scale for amino acids is used to indicate sequence similarity; Figure S8: Tissues in which the CLSY1-4, AT2G16390/DRD1, and AT2G21450 genes are most expressed in *Arabidopsis*. The scale in the Shoot Apex column refers to the expression of genes in shoot apex, seed, and developing seed tissues. The scale in the carpel column refers to the expression of genes in carpel, young seeds, and flower tissues; Figure S9: Expression profile of *Arabidopsis* genes CLSY1-4, AT2G16390/DRD1, and AT2G21450 under nine types of abiotic stresses in shoot and root tissues. (a) CLSY1; (b) CLSY2; (c) CLSY3; (d) CLSY4; (e) AT2G16390/DRD1; (f) AT2G21450; Figure S10: Expression profiles of soybean genes from the three clades in 14 different tissues. (a) Clade 1 genes; (b) Clade 2 genes; (c) Clade 3 genes. No results were reported for the gene Glyma.02G261800; Figure S11: Relative expression of Glyma.U027200 and Glyma.08G339900 genes in embryonic axes of cultivar BRS284, which refers to the same transcriptome used in the Soybean Expression Atlas database. The asterisk indicates significantly different expression values between genes according to the t-test (*p* < 0.05); File S1: Profile HMMs specifically constructed for CLSY detection.

## Abbreviations

The following abbreviations are used in this manuscript:

AGO	ARGONAUTE
CLSY	CLASSY
DCL3	DICER-LIKE 3
DRD1	DEFECTIVE IN RNA DIRECTED DNA METHYLATION 1
DRM2	DOMAINS REARRANGED METHYLTRANSFERASE 2
dsRNA	double-stranded RNA
HMM	Hidden Markov models
Pol	RNA Polymerase
RdDM	RNA-directed DNA methylation
RDR2	RNA-DEPENDENT RNA POLYMERASE 2
ROS1	REPRESSOR OF SILENCING 1
RT-qPCR	Reverse transcription-quantitative polymerase chain reaction
SHH1	SAWADEE HOMEODOMAIN HOMOLOG 1
siRNA	small interfering RNA
sRNA	small RNA
ssRNA	single-stranded RNA
TE	Transposable element
WGD	Whole-genome duplication
