# Supplementary material for: RdDM-Associated Chromatin Remodelers in Soybean: Evolution and Stress-Induced Expression of CLASSY Genes
Source: Plants (Basel). 2025 Aug 15;14(16):2543. doi: 10.3390/plants14162543 (PMC12389316; doi:10.3390/plants14162543)
Supplement: Supplementary file 1 [file plants-14-02543-s001.zip › Supplementary materials.pdf]

Supplementary materials

Table S1. Primer sequences (forward/reverse) used in RT-qPCR analysis.

Gene name	Sequence (5′-3′)
Glyma.U027200_F	CAAGAGCATCAATAAGGGACG
Glyma.U027200_R	CAAACCGAAAGCAAACCTCC
Glyma.08G339900_F	TGTGGGCTGAAATGGAGATG
Glyma.08G339900_R	CACTGCAAGGCTGGATTTTC
AGO1_F	CAAAAGCGTCACCACACAAG
AGO1_R	AGTCAAATTCGGTGGGATGG
AGO4_F	TCTCGATGAAAATTGGGAGCC
AGO4_R	TGTCGATAACAGTTCCAGGTG
DCL3_F	GCCAGTGCCTACATTTGATTC
DCL3_R	TTCTGTCCCTTGCTTCCTTG
DRM2_F	CGGTCTTTGGTGAAAATGGG
DRM2_R	CTTCAGCCTTTGCCATTTGAG
ROS1_F	GCCAACAGAGACAAATGAGC
ROS1_R	CCTAATCTGGAAAAACTGCTCAG
METK4_F	CTGAGCCTTTGTCTGTGTTTG
METK4_R	CCTGTTATTTCCACCCCTCTTG

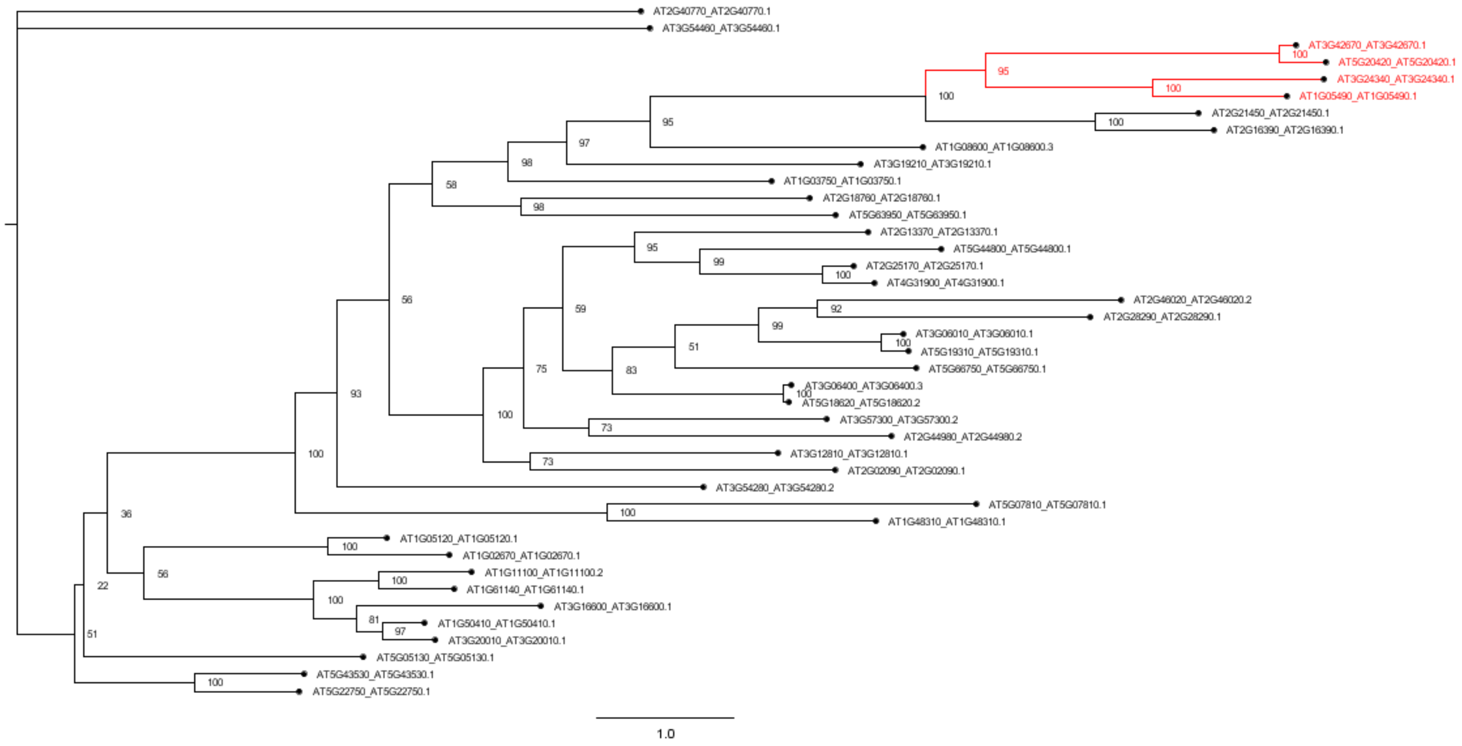

Figure S1. Phylogenetic tree of 41 proteins containing the SNF2 domain. *Arabidopsis* CLSY1-4 proteins are highlighted in red. The values showed on the branches represent the bootstrap values from 1000 replicates.

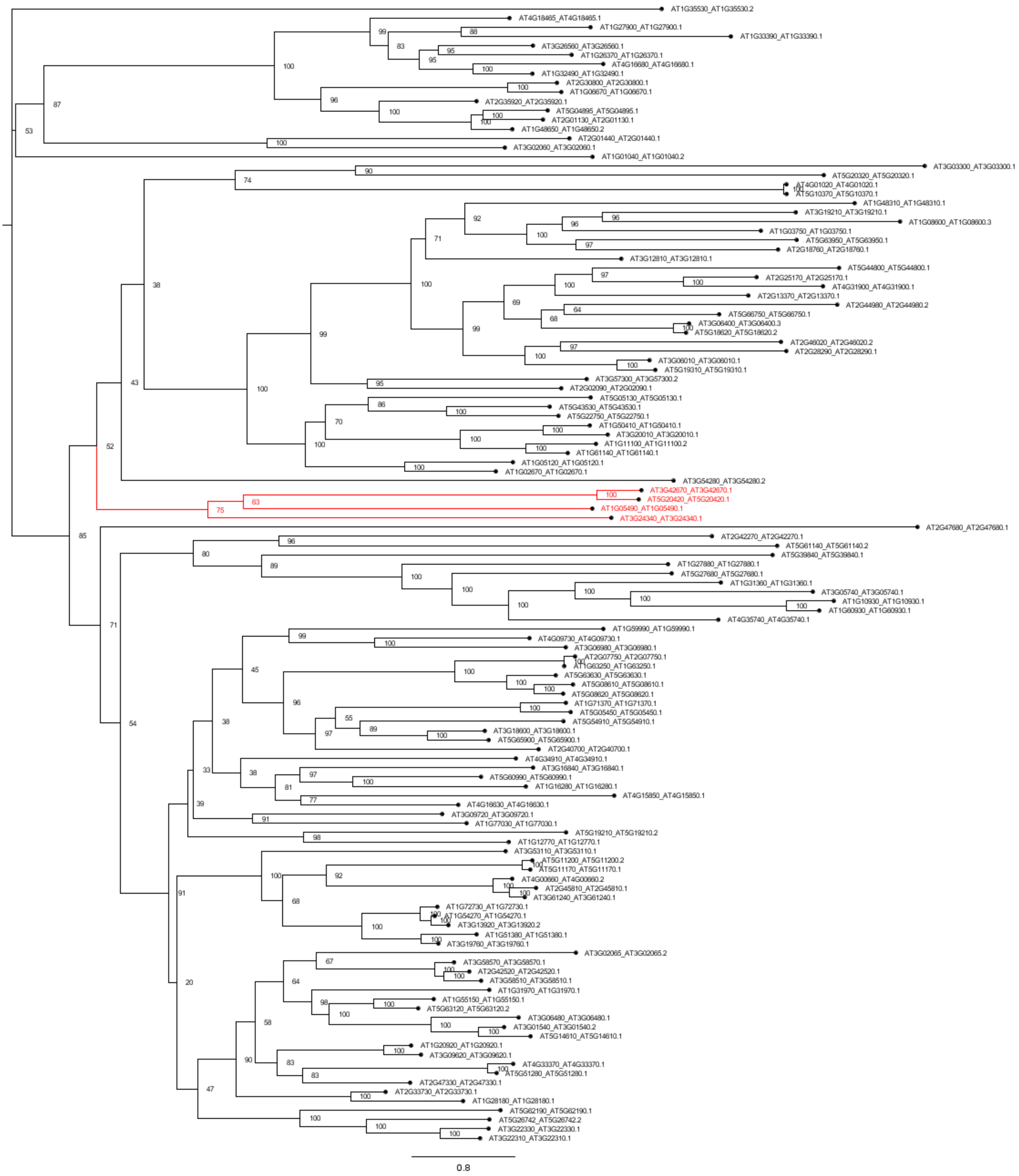

Figure S2. Phylogenetic tree with the 123 proteins featuring the Helicase C domain. *Arabidopsis* CLSY1-4 proteins are highlighted in red. The values showed on the branches represent the bootstrap values from 1000 replicates.

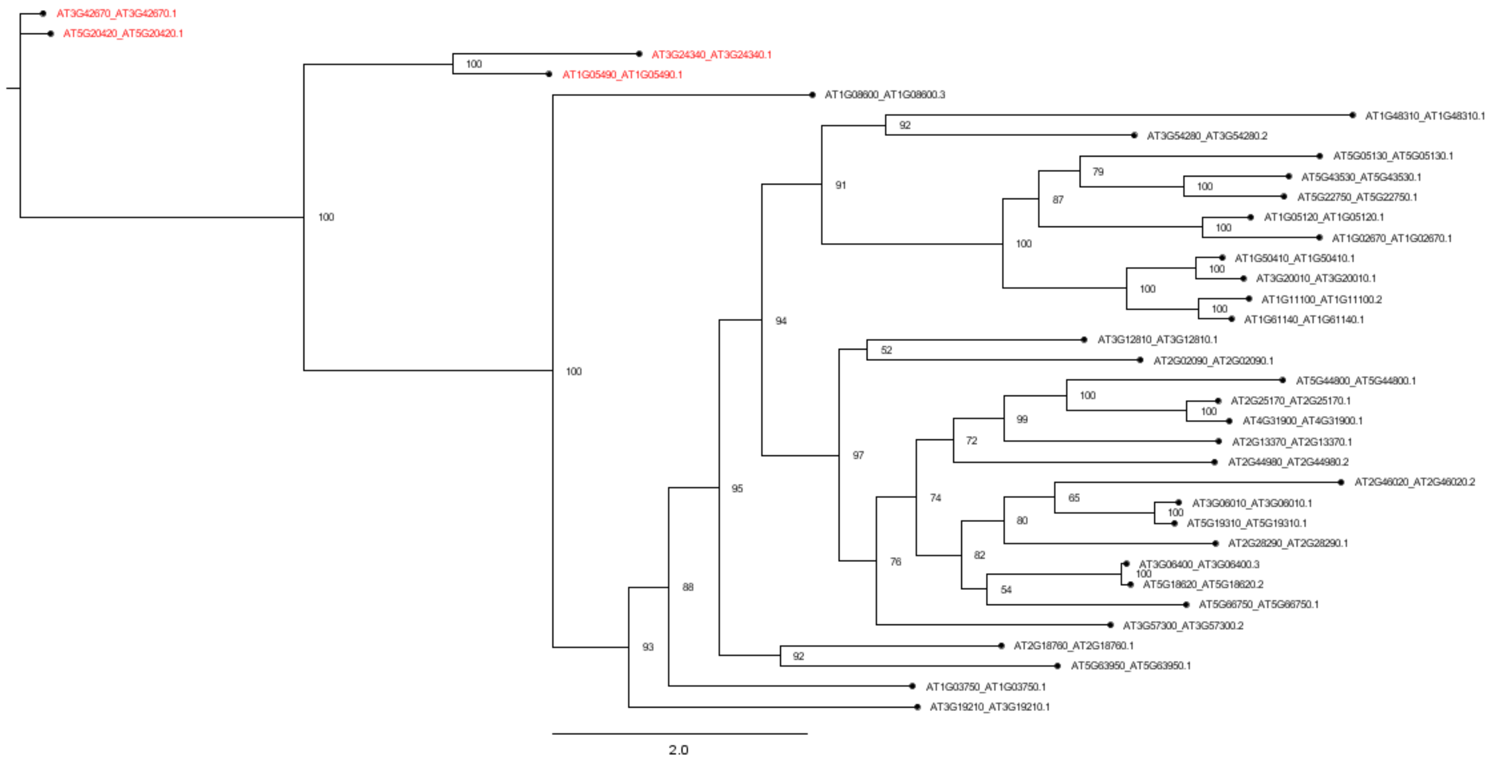

Figure S3. Phylogenetic tree with the 35 proteins in common between the SNF2 and Helicase C domains. *Arabidopsis* CLSY1-4 proteins are highlighted in red. The values showed on the branches represent the bootstrap values from 1000 replicates.

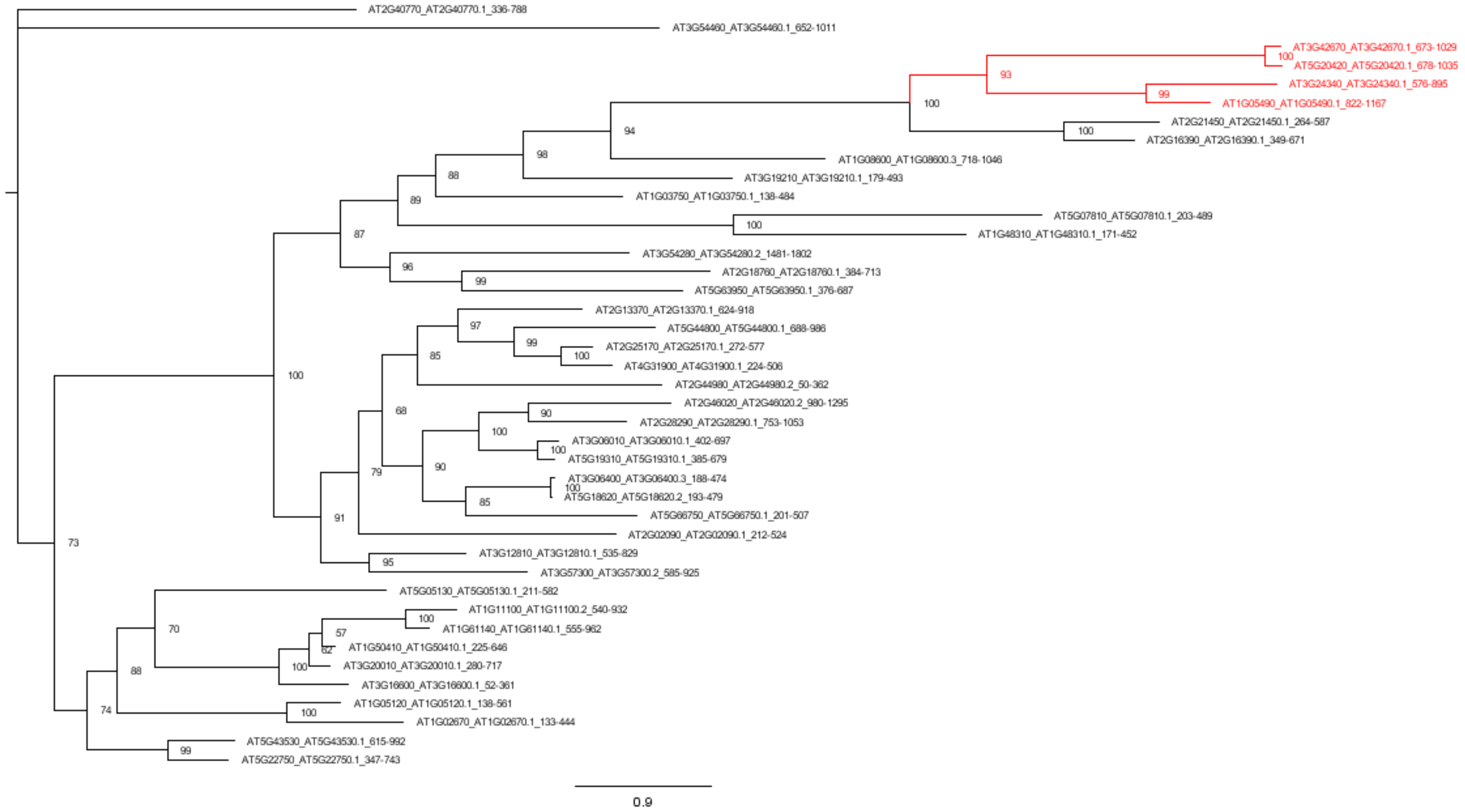

Figure S4. Phylogenetic tree containing the 41 SNF2 domains, but excluding the Helicase C domain of the 35 proteins with both domains. *Arabidopsis* CLSY1-4 proteins are highlighted in red. The values showed on the branches represent the bootstrap values from 1000 replicates.

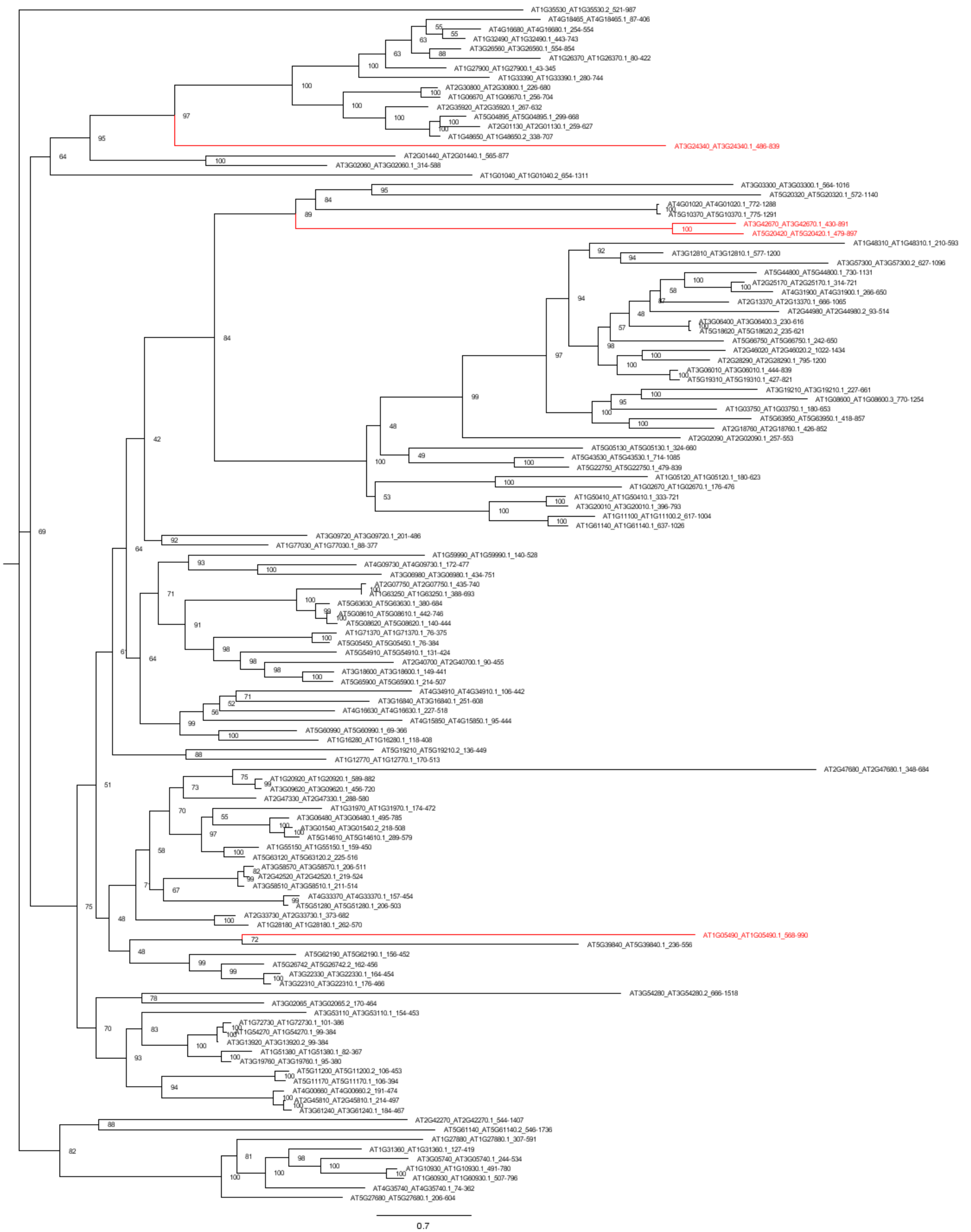

Figure S5. Phylogenetic tree comprising the 123 Helicase C domains, but excluding the SNF2 domain of the 35 proteins. *Arabidopsis* CLSY1-4 proteins are highlighted in red. The values showed on the branches represent the bootstrap values from 1000 replicates.

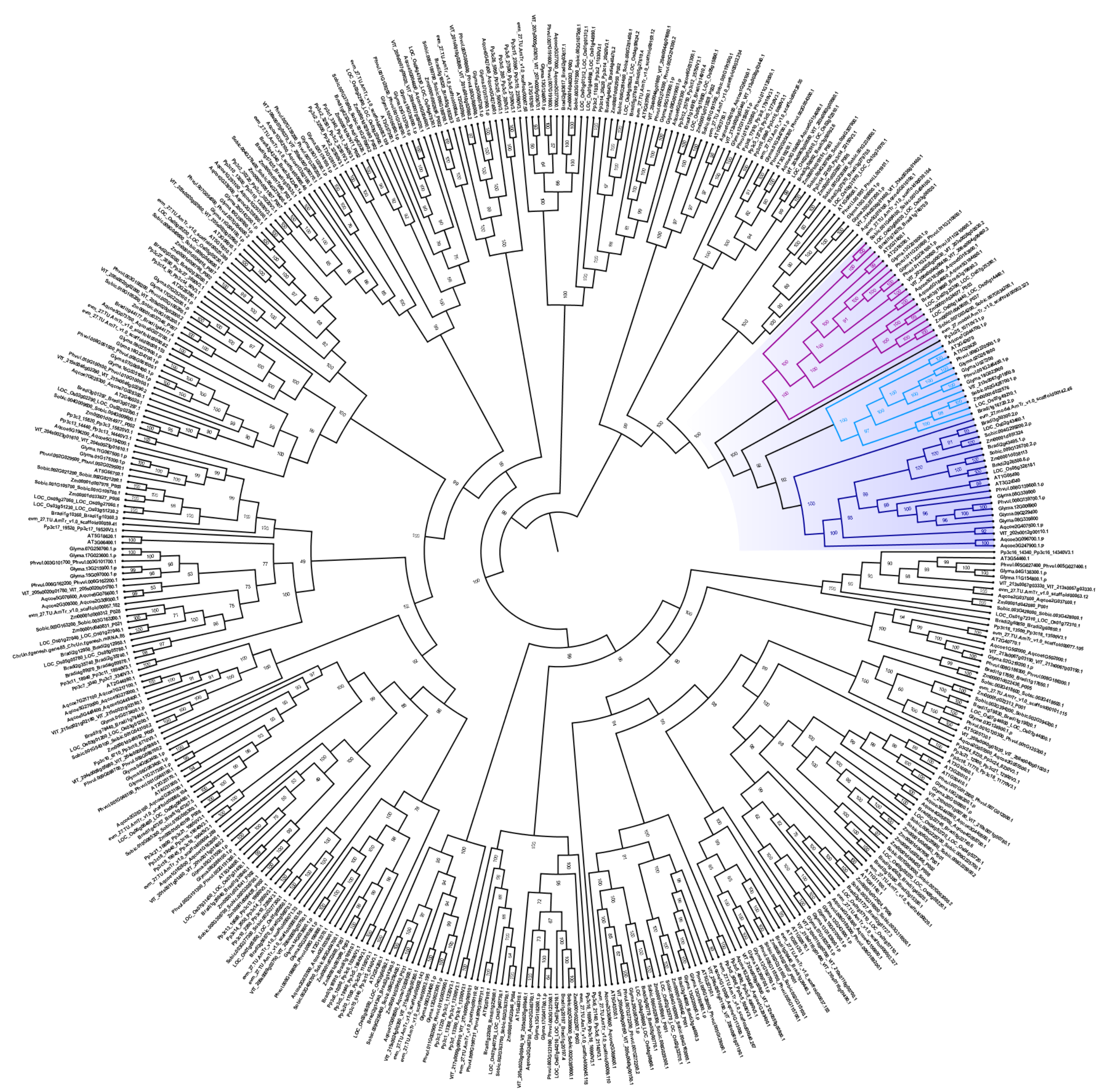

Figure S6. Complete phylogenetic tree of the 447 identified proteins, with emphasis on the monophyletic group containing proteins related to the CLSY and DRD1 families. Clades 1, 2, and 3 are shown in light blue, dark blue, and purple, respectively. The values indicated on the branches represent the bootstrap values from 1000 replicates.

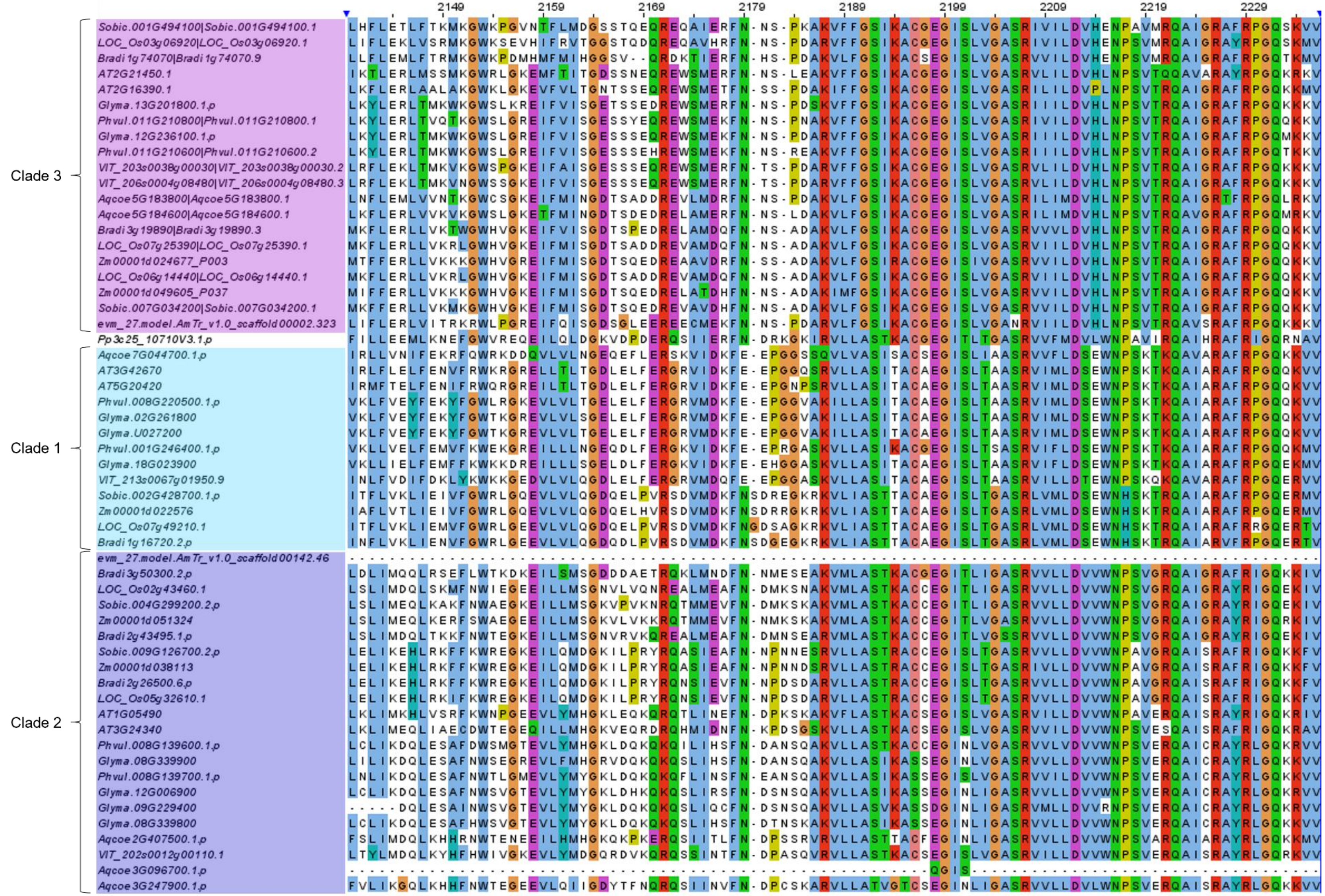

Figure S7. Multiple sequence alignment of the Helicase C domain obtained in Jalview, highlighting the clades 1, 2, and 3. The clades are in the order in which they were arranged in the phylogenetic tree (Figure S6). A color scale on amino acids was used to indicate sequence similarity.

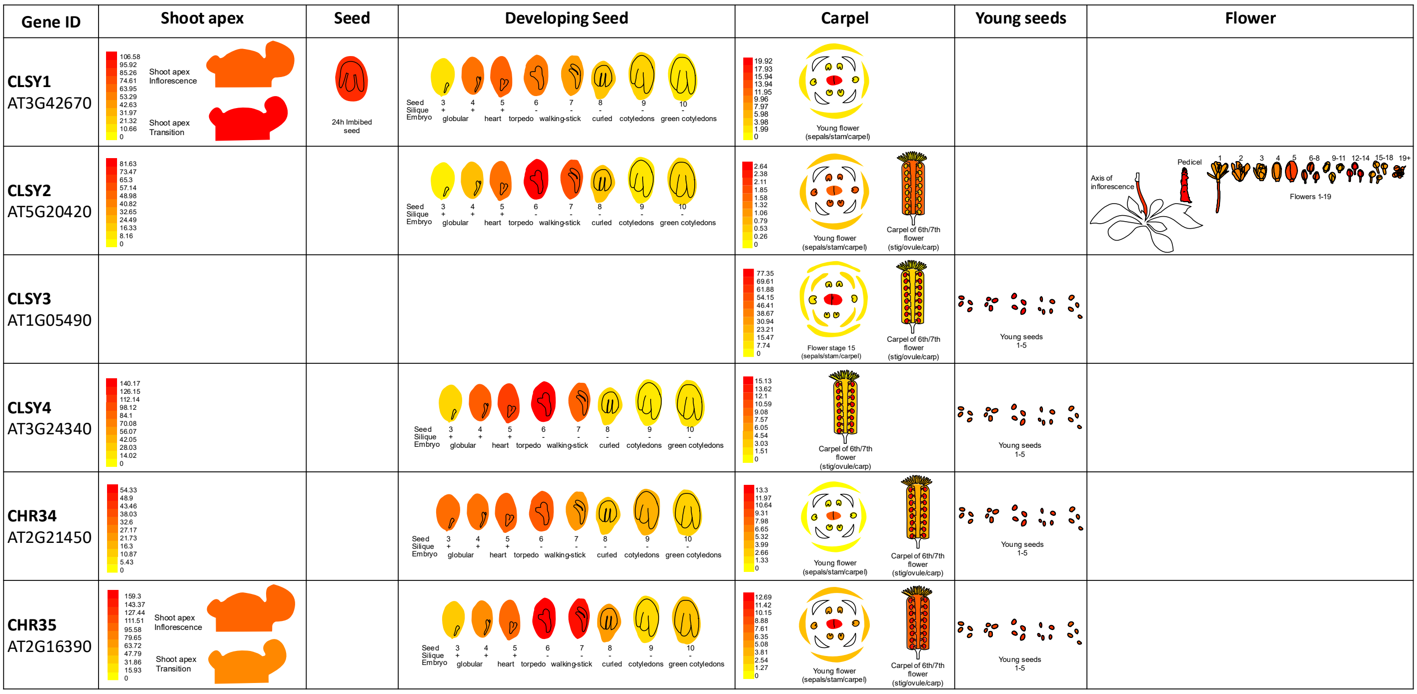

Figure S8. Tissues in which the CLSY1-4, AT2G16390/DRD1, and AT2G21450 genes are most expressed in *Arabidopsis*. The scale in the Shoot Apex column refers to the expression of genes in shoot apex, seed, and developing seed tissues. The scale in the carpel column refers to the expression of genes in carpel, young seeds, and flower tissues.

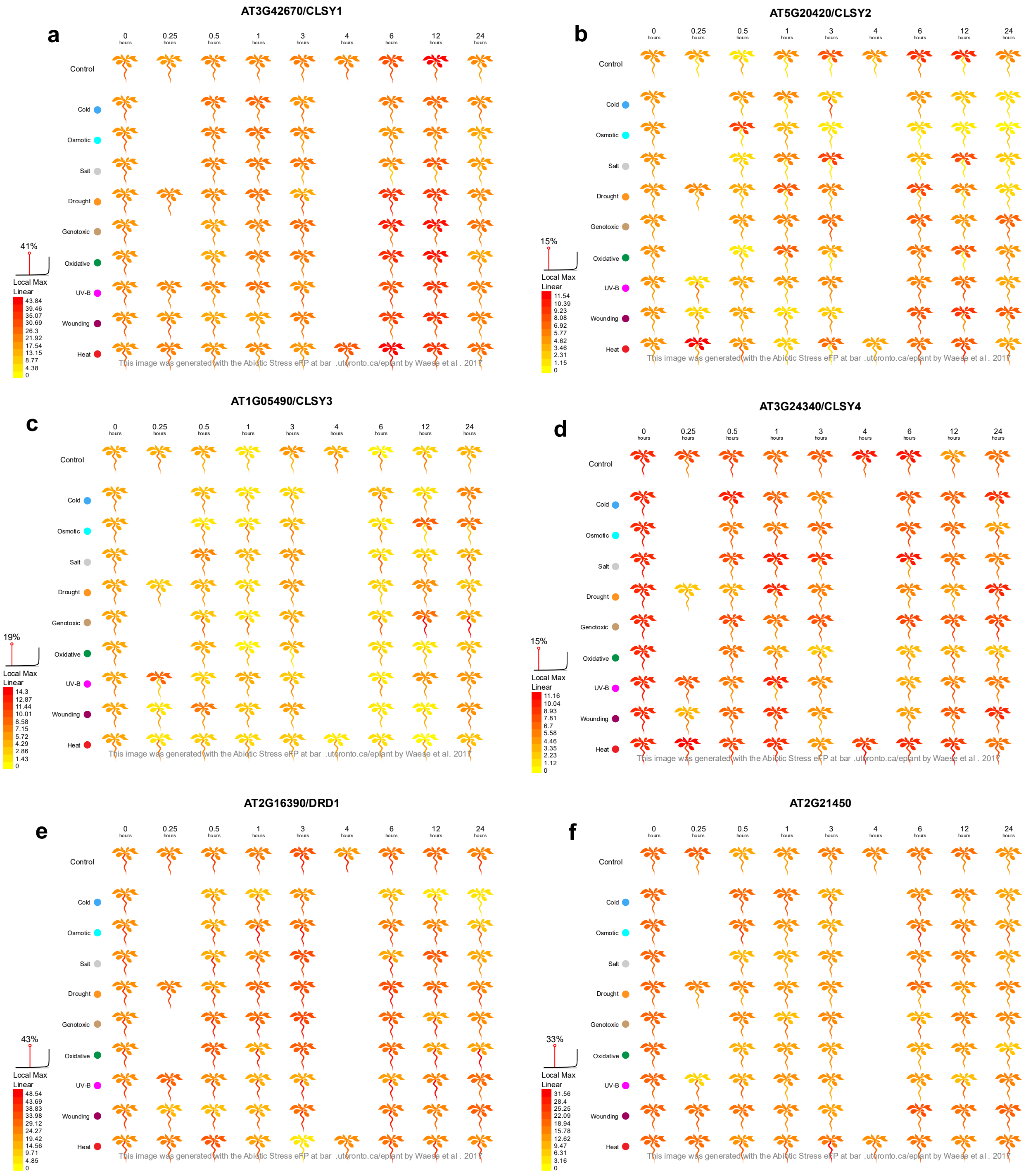

Figure S9. Expression profile of *Arabidopsis* genes CLSY1-4, AT2G16390/DRD1, and AT2G21450 under nine types of abiotic stresses in shoot and root tissues. (a) CLSY1; (b) CLSY2; (c) CLSY3; (d) CLSY4; (e) AT2G16390/DRD1; (f) AT2G21450.

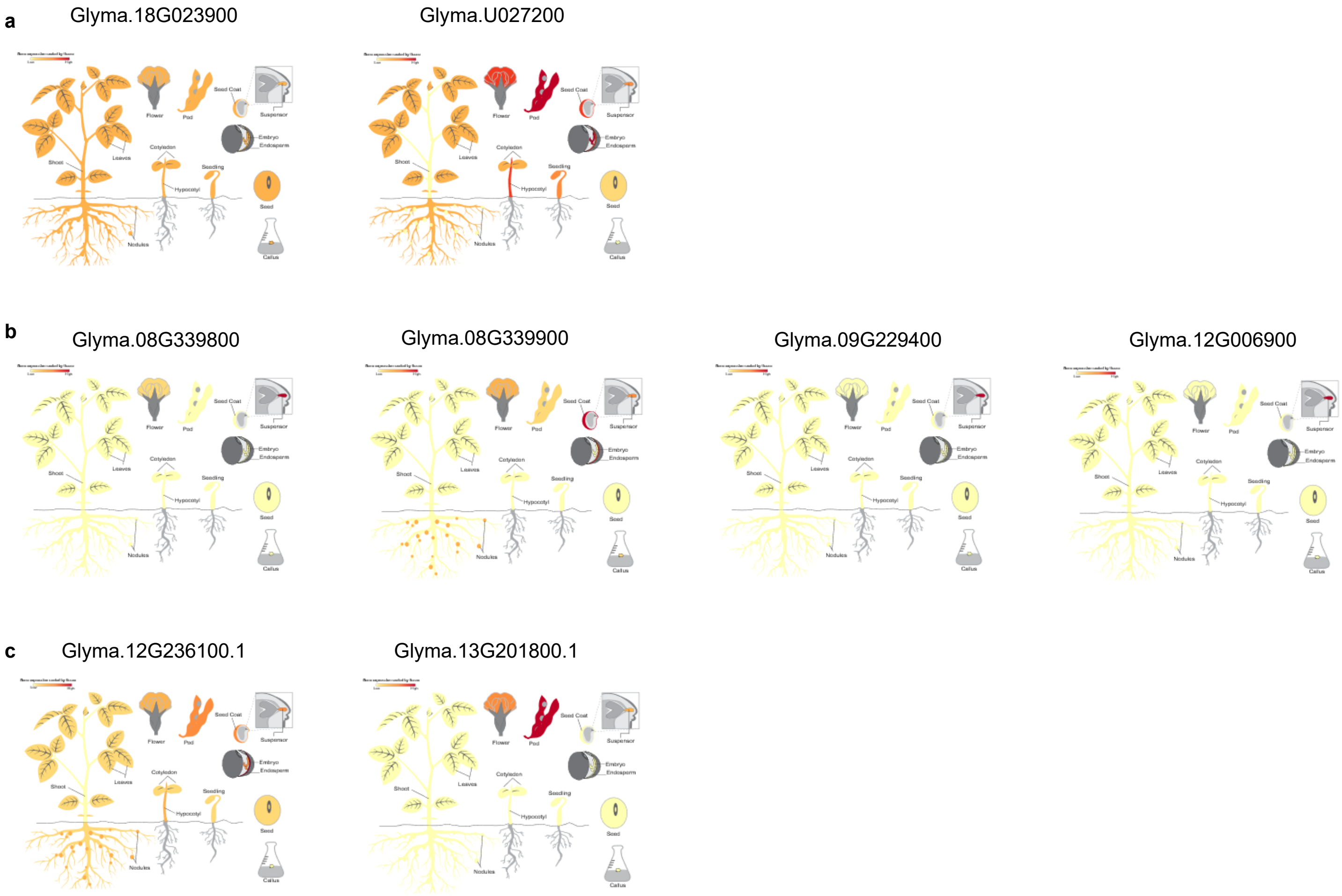

Figure S10. Expression profiles of soybean genes from the three clades in 14 different tissues. (a) Clade 1 genes; (b) Clade 2 genes; (c) Clade 3 genes. No results were reported for the gene Glyma.02G261800.

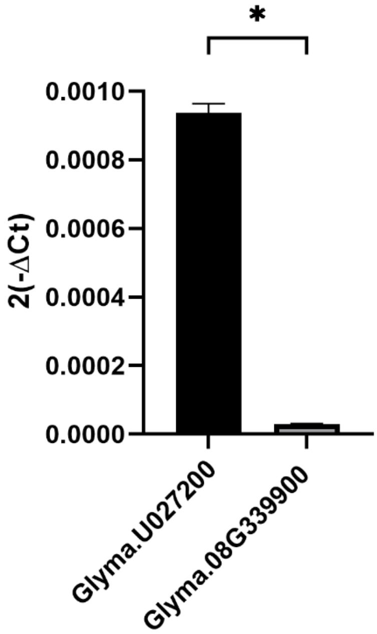

Figure S11. Relative expression of Glyma.U027200 and Glyma.08G339900 genes in embryonic axes of cultivar BRS284, which refers to the same transcriptome used in the Soybean Expression Atlas database. The asterisk indicates significantly different expression values between genes according to the t-test ($P < 0.05$).
